# Distress Calls of a Fast-Flying Bat (*Molossus molossus*) Provoke Inspection Flights but Not Cooperative Mobbing

**DOI:** 10.1371/journal.pone.0136146

**Published:** 2015-09-09

**Authors:** Gerald Carter, Diana Schoeppler, Marie Manthey, Mirjam Knörnschild, Annette Denzinger

**Affiliations:** 1 Biology Department, University of Maryland, College Park, MD, United States of America; 2 Animal Physiology, Institute for Neurobiology, University of Tuebingen, Tuebingen, Germany; 3 Institute of Evolutionary Ecology and Conservation Genomics, University of Ulm, Ulm, Germany; 4 Smithsonian Tropical Research Institute, Balboa, Panama; University of Western Ontario, CANADA

## Abstract

Many birds and mammals produce distress calls when captured. Bats often approach speakers playing conspecific distress calls, which has led to the hypothesis that bat distress calls promote cooperative mobbing. An alternative explanation is that approaching bats are selfishly assessing predation risk. Previous playback studies on bat distress calls involved species with highly maneuverable flight, capable of making close passes and tight circles around speakers, which can look like mobbing. We broadcast distress calls recorded from the velvety free-tailed bat, *Molossus molossus*, a fast-flying aerial-hawker with relatively poor maneuverability. Based on their flight behavior, we predicted that, in response to distress call playbacks, *M*. *molossus* would make individual passing inspection flights but would not approach in groups or approach within a meter of the distress call source. By recording responses via ultrasonic recording and infrared video, we found that *M*. *molossus*, and to a lesser extent *Saccopteryx bilineata*, made more flight passes during distress call playbacks compared to noise. However, only the more maneuverable *S*. *bilineata* made close approaches to the speaker, and we found no evidence of mobbing in groups. Instead, our findings are consistent with the hypothesis that single bats approached distress calls simply to investigate the situation. These results suggest that approaches by bats to distress calls should not suffice as clear evidence for mobbing.

## Introduction

“Distress calls” are produced by captured or trapped animals of many species, including frogs [1], lizards [2], crocodilians [3], birds [4–9], bats [10–17], and other mammals [18–22]. In primates, distress calls are produced by all age categories, sexes, and species in which vocal communication has been studied [17, 22]. Because primate distress calls will often solicit help from groupmates, distress call playbacks have been used to test social knowledge and social relationships [e.g. 20–21]. In contrast, it is still unclear whether distress calls even serve a social function in most other species. Authors have proposed a variety of non-mutually exclusive hypotheses for why distress calls might be adaptive [23]. For example, they might altruistically warn kin of danger or they might aid in escape by startling the predator, by attracting conspecifics, or by luring secondary predators to scare away the attacking predator [23]. Alternatively, distress calls might simply be a non-adaptive but non-costly byproduct of an ancestral trait.

Conspecifics and heterospecifics often approach the source of distress calls reliably enough to make them suitable as acoustic lures [5,10–16]. Several authors have suggested that the strong attraction of other bats to distress calls indicates that the function of these vocalizations is to evoke cooperative mobbing [11–15, 18], an antipredator behavior whereby groups of animals attack, harass, or approach potential predators to deter them.

There are however several reasons to be skeptical of the notion that distress calls function to provoke mobbing in bats. First, although cooperative mobbing is found in many social birds and some mammals, and sometimes triggered by alarm or mobbing calls [19, 24–31], these calls are typically distinct in structure and function from distress calls. Second, prey animals might merely approach potential predators to gather information [32–34]. Third, direct observational evidence for mobbing in bats has only been reported twice; greater-spear nosed bats *Phyllostomus hastatus* and naked-bellied tomb bats *Taphozous nudiventris* have each been observed harassing an owl [30–31]. This paucity of observations might simply result from the nocturnal flight behavior of bats being difficult to observe, but it might also accurately represent the rarity of cooperative mobbing by bats in nature.

A simpler explanation for why bats approach distress calls is that they are individually and selfishly investigating the situation. All bat species known to be attracted to distress call playbacks are highly maneuverable fliers capable of making tight swooping flights [10–16]. In these species, inspection flights can easily look like mobbing behavior because scanning with nocturnal vision or echolocation requires a closer approach than vision in daylight. We tested response to distress calls in a fast-flying, aerial-hawking bat with relatively poor maneuverability: the velvety free-tailed bat *Molossus molossus*. This species is a group-living and possibly group-hunting [35] insectivore that is highly adapted for hunting in open spaces, as reflected by its wing morphology [36, 37] and an echolocation call design that allows for long-range detection [38–40]. Given their relatively poor maneuverability, we therefore asked: Would playbacks of *M*. *molossus* distress calls attract groups of conspecifics to within one meter of the speaker, close enough to harass a predator? Or would *M*. *molossus* respond with passing investigative flights that maintain a safe distance? We also examined whether *M*. *molossus* would produce social calls more often during distress call playback than during controls.

On Barro Colorado Island, Panama where we conducted our study, several groups of *M*. *molossus* roost in the laboratory buildings and exit their roosts quickly using distinct flight paths. When returning from the foraging sites, bats often fly in the vicinity of the roost for some time before entering. Several groups of greater sac-winged bats (*Saccopteryx bilineata*) also live on the outside of the same buildings. This species departs earlier and forages nearby, is highly maneuverable and able to hover [41], and is also attracted by the distress calls of conspecifics (unpublished data). We therefore also tested if *S*. *bilineata* would be attracted by the heterospecific distress calls of *M*. *molossus*, and if so, whether their approaches would be closer to the speaker as predicted by their flight abilities.

Lastly, we investigated if the responsiveness of *M*. *molossus* to distress calls would increase over time. At our study site, *M*. *molossus* depart from their roosts at sunset, forage intensely for only ~50 min, then return to their roosts [42]. If bats respond to distress calls in order to mob predators, then we predicted that *M*. *molossus* might be less responsive to distress calls before foraging, and would be increasingly responsive to distress calls as the night progressed. This prediction is based on the assumption that the the expected fitness costs to an individual mobbing bat would be greater at emergence when their energy reserves are lower and avian predators would have more light. On the other hand, if the approaches do not pose a significant cost or risk, then we do not expect a difference in responsiveness over time.

## Methods

### Ethics statement

This study was approved by the Institutional Animal Care and Use Committee of the University of Maryland College Park (IACUC Ref#: R-14-07) and of the Smithsonian Tropical Research Institute (IACUC Ref# 2013-1015-2016 and 2014-0815-2017).

### Recording and measuring distress calls

**Fig 1:**
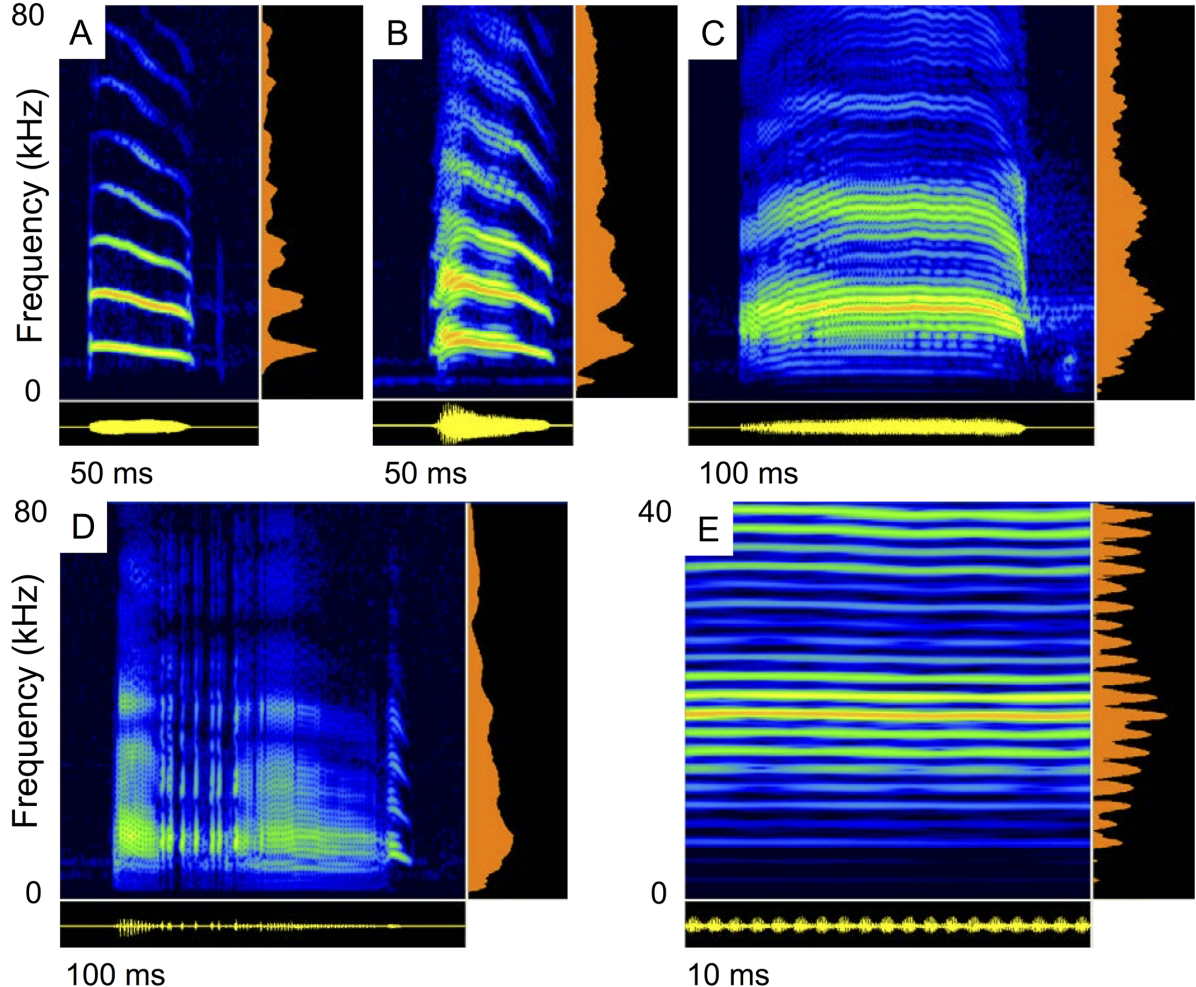
Variation of distress calls of *Molossus molossus*. Spectrograms (FFT 1024, Blackman, auto padding, dynamic range of 90 dB) of distress calls playbacks with averaged power spectrum (aside) and waveform (below). Distress calls structures included multiharmonic shallowly modulated calls (A), calls with sideband modulations (B, C), and some calls with nonlinear phenomena (D). Sideband modulations of the distress call shown in C are depicted in E (FFT 2048).

We located eight roosts of *Molossus molossus* in the laboratory buildings at the Smithsonian Tropical Research Station at Barro Colorado Island, Panama (9°9´17´´ N, 79°51´53´´ W). We recorded distress calls (Fig 1) from 9 adult male *M*. *molossus* that were entangled in mist-nets (Ultrathin Mist Nets M-14; Ecotone, Gdynia, Poland) at ~1 m distance with a handheld USG 116Hm (Avisoft Bioacoustics, Berlin, Germany, Avisoft.com; gain set to 0 dB, sample rate 500 kHz, frequency response: 5–30 kHz ± 4dB, 30–100 kHz ± 2dB). We analyzed the *M*. *molossus* distress calls used in our playback sequences via color spectrograms (FFT 512, Blackman, dynamic range 90 dB) and waveforms displayed with the software Selena (Animal Physiology, University of Tuebingen). Call duration and call interval (time between the onsets of two consecutive calls) were measured from waveforms. Upper and lower frequency limits were set 25 dB below maximum amplitude. Sideband modulations [43] were measured (FFT 2048) in five calls per bat.

### Constructing playback sequences

**Fig 2:**
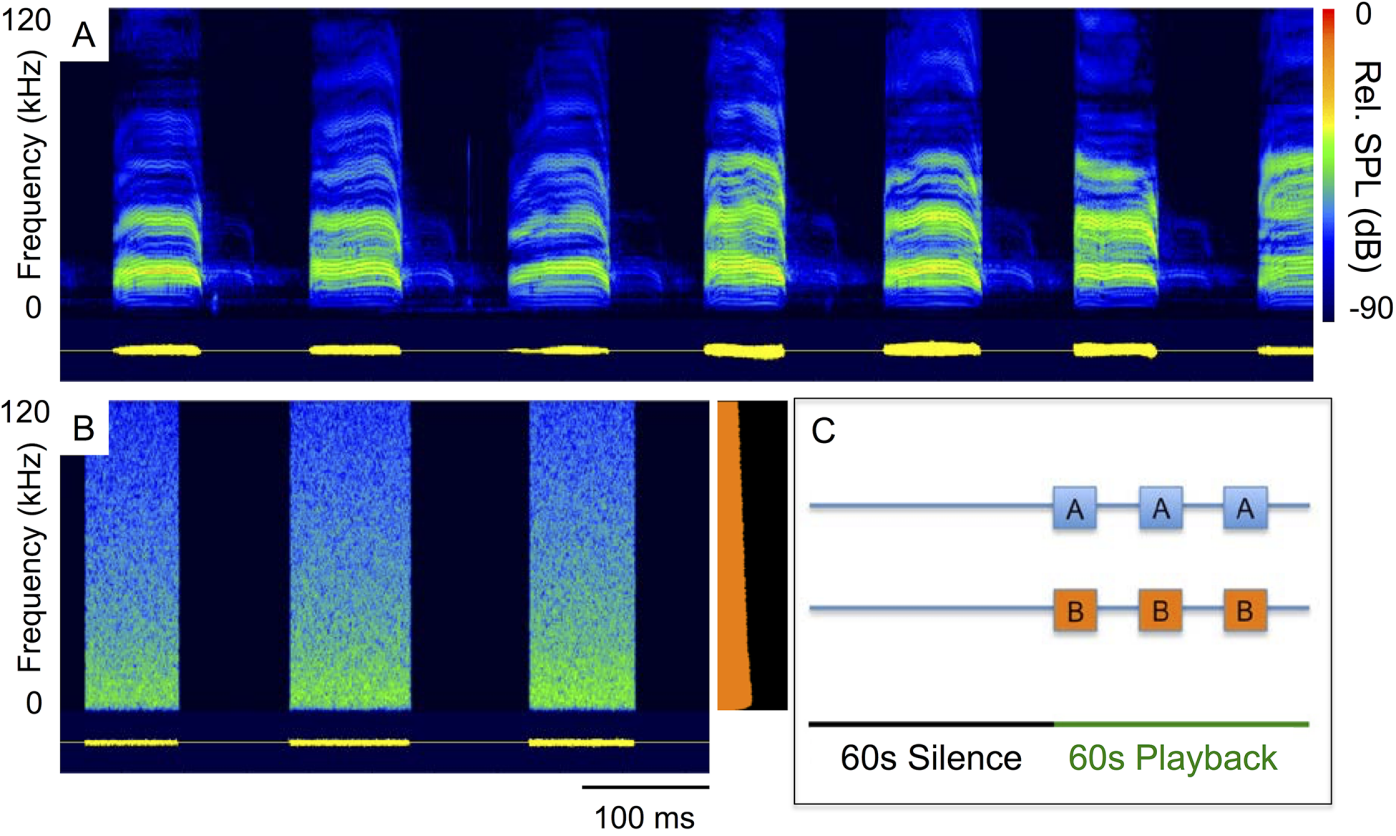
Distress calls and pink noise stimuli. Spectrograms (500 kHz sampling rate, 16 bit resolution, 1024 FFT, Blackman window) of a sequence of *Molossus molossus* distress calls (A) and pink noise controls (B) with averaged power spectrum of the third pink noise stimulus. Each 2-min playback sequence (C) begins with a 1-min silent period followed by a 1-min playback period with either distress calls (A boxes) or pink noise bursts (B boxes) repeated three times and spaced apart by 10 s of silence.

Using BatSound Pro (Pettersson Elektronik, Uppsala, Sweden), we constructed 12 unique 10 s clips of distress calls that preserved the spacing in the original distress call recordings (Fig 2A). Different calls of one male were used for two clips, different calls of a second male were used for three clips and the remaining seven males contributed to one clip each. Each clip included 35–60 calls.

For each distress call clip, we created a paired pink noise control clip that roughly matched the variation in amplitude and temporal spacing of each distress call clip (Fig 2B). We then expanded each 10 s clip (12 distress and 12 paired noise clips) into a 120-s playback sequence (Fig 2C) consisting of 60 s of silence (“silent period”) followed by a 60 s period with three repeats of 10 s silence and the 10 s distress call or noise clip (“playback period”).

To eliminate background noise, we bandpass-filtered all the sequences at 2.5–200 kHz (Butterworth filter, filter order = 2). To equalize and maximize signal strength, we adjusted the amplitude of signals in Batsound Pro so that the highest amplitude signal was near 100% without being clipped (adjustments ranged from 1-3x the original).

We measured the SPL range of playbacks to be 77–94 dB SPL rms re to 20 μPa at 1m. We obtained these measures by recording the playback of three paired exemplar distress call and noise stimuli (lowest, highest, and 50% relative amplitude) using a 1/8 inch precision pressure microphone (40DP, G.R.A.S. Sound & Vibration, Denmark, gras.dk) linked to a 12AK Power module (G.R.A.S.) at a distance of 23 cm in a room covered in acoustic foam, and then measured peak-to-peak amplitude (mV) on a Tektronix TDS2014 digital oscilloscope (Tektronix, Inc., tek.com).

### Experimental design

We recorded bat passes while broadcasting playbacks during 12 playback sessions on 8 nights at 8 different sites at the Barro Colorado Island field station. Each playback session included 12 unique distress call playback sequences and 12 unique noise playback sequences in alternating order (e.g. distress, noise, distress, noise, etc) spaced apart by at least 60 s of silence. Playback sequences in each session were looped so that the first playback sequence began again after the last. Equipment errors and weather led to some playbacks being interrupted, so playback sessions included 6–50 playback sequences (12 min–100 min).

In total, we broadcast 308 playback sequences (155 and 153 distress and noise playbacks respectively) using an Avisoft USG Player BL Pro speaker (frequency response: 5–80 kHz ± 4 dB). We simultaneously recorded continuously at constant gain with an Avisoft CM16 microphone connected to an UltrasoundGate 116Hn (500 kHz sampling rate, 16 bit resolution) that was pointed in the same direction as the speaker such that bat calls directed towards the speaker would be highest in amplitude. By visual inspection of the spectrograms, we could easily discriminate between our playbacks and real bat calls.

### Measuring bat responses

We defined a “pass” as one or more echolocation calls where at least one call exceeded an amplitude threshold of 10% in BatSound Pro. A typical single pass consists of a series of echolocation pulses that begin below 10% amplitude, increase over 10%, then decreases again below 10%. This pattern indicates that a bat was flying nearby the speaker or turning towards it.

By examining the spectrograms, we categorized each pass as *Molossus* sp. (probably *M*. *molossus*), *Saccopteryx bilineata* or “other” (usually *Myotis nigricans*). We assumed that *Molossus* calls we recorded were *M*. *molossus*, because this species was by far the most abundant molossid bat in the area: more than 95% of molossid bats (n = 256) caught in the areas where we conducted our experiments were *M*. *molossus*, and others were *M*. *sinaloae* or *M*. *bondae* (unpublished data 2010–2014).

We measured two responses in *M*. *molossus* and *S*. *bilineata*. “Activity” is the number of bat passes occurring during the 2-min playback sequence. “Responsiveness” is the change in the number of passes after onset of playback (i.e., number of passes during the 1-min playback minus the number of passes during previous 1-min silence; Fig 2C). Additionally, we scored if a *M*. *molossus* pass included a social call.

To observe close approaches to the speaker, we illuminated and videotaped the speaker using an infrared (IR) spotlight (IRLamp6, Wildlife Engineering, irlight.com) and a Sony DCR-SR85 Nightshot camcorder. This allowed us to observe an area of more than a square meter in front of the speaker. We reviewed video footage in fast-forward during periods with no recordings detected, and carefully reviewed footage in real time or slower during periods with an increase of 3 passes during the 1-min playback (responsiveness > 3 passes).

### Statistical analysis

We tested (1) if the number of bat passes increased from the 1-min silent period to the 1-min playback period (mean responsiveness > 0) and (2) if this increase in bat passes (responsiveness) was greater during distress call playback compared to noise playback. We inspected histograms and normal quantile plots to confirm normality of responsiveness values. To test the effect of playback treatment on responsiveness, we used restricted maximum likelihood to fit a linear mixed model in JMP 12 [44] with playback treatment (distress or noise) as a fixed factor and playback sequence (1–12) as a random factor nested within treatment.

We excluded observations of zero bat passes during both the silent and playback period, because these cases indicated an absence of bats, and a zero-inflated dataset would inflate sample size, reduce effect size estimates, and create deviation from normality. To check the robustness of our results, we also repeated our analyses including these cases with zero bat passes, using a permuted linear model (lmPerm package in R) for inference. We only present the analysis excluding the sequences with no bat passes present, but our conclusions were the same using either approach.

To determine if *M*. *molossus* responsiveness to distress calls differed between when bats were either departing or returning to roosts, we first plotted *M*. *molossus* activity and responsiveness during distress call sequences over time. We analyzed recordings with bats present from four evenings (n = 54 playback sequences; 108 min) where the microphone and speaker were positioned within ~20 meters of a roost but facing in such a way that the bats would need to deviate from their normal flight paths to approach the speaker. We then tested if *M*. *molossus* activity or responsiveness increased from emergence (1840 h) until return (2000 h) by fitting a linear model in JMP 12.

## Results

### Distress call variation

Distress call structure from the nine male *Molossus molossus* varied both within and among individuals (Fig 1), from pure multiharmonic shallowly modulated signals to calls with nonlinear phenomena. Most distress calls had sideband modulations [43] (Fig 1E). The mean modulation frequency was 1.7 kHz. The mean overall bandwidth set by the upper and lower frequency limits was 45 kHz with a mean lower frequency limit of 10 kHz. Average call duration was 64 ms and call interval ranged from 0.06 to 1.3 s (Table 1). The peak frequency corresponded to the first harmonic in 90% of the calls (n = 506; mean peak frequency = 16.0 kHz ± 3.6 kHz S.E.), to the second harmonic in 9% of calls (n = 51; mean = 32.9± 7.1 kHz), and to the third harmonic in 1% of calls (n = 4; mean = 50.9 ± 9.6 kHz).

**Table 1:** Call parameters of playback distress calls (mean ± standard deviation).

Bat	N	Call duration(ms)	Call interval (ms)	Peak freq. (1^st^ harm.) (kHz) (N)	Max freq (kHz)	Min freq (kHz)	Bandwidth (kHz)	Freq. of sideband modulation (kHz)
1	111	73.1 ± 10.9	179.0 ± 39.6	18.4 ± 1.7 (103)	12.5 ± 1.7	61.3 ± 10.6	48.9 ± 10.9	2.0 ± 0.2
2	145	54.1 ± 16.6	207.7 ± 140.5	15.2 ± 3.2 (141)	9.1 ± 1.6	58.0 ± 10.5	48.9 ± 10.8	2.0 ± 0.1
3	60	50.3 ± 15.0	164.1 ± 26.9	12.0 ± 1.1 (58)	8.1 ± 1.5	39.2 ± 8.7	31.2 ± 9.1	1.4 ± 0.1
4	18	63.0 ± 18.1	263.1 ± 227.8	16.8 ± 6.5 (17)	7.9 ± 3.4	52.7 ± 3.4	44.9 ± 8.6	1.6 ± 0.1
5	39	65.1 ± 14.7	248.7 ± 145.8	17.9 ± 4.6 (32)	9.7 ± 3.0	49.8 ± 7.7	40.1 ± 7.8	1.9 ± 0.2
6	41	68.3 ± 27.9	241.3 ± 129.1	16.3 ± 4.3 (41)	9.9 ± 2.3	56.1 ± 8.6	46.2 ± 10.0	1.8 ± 0.3
7	54	72.5 ± 15.8	183.2 ± 35.2	16.8 ± 4.2 (36)	11.3 ± 2.4	58.9 ± 9.0	47.6 ± 9.2	1.9 ± 0.1
8	44	65.9 ± 25.5	225.1 ± 179.0	19.9 ± 1.5 (32)	10.3 ± 3.8	50.7 ± 11.0	40.4 ± 11.1	1.6 ± 0.1
9	49	75.1 ± 14.9	206.1 ± 39.2	15.6 ± 2.1 (46)	9.2 ± 2.8	56.5 ± 9.1	47.3 ± 9.7	1.6 ± 0.0
$\bar{X}$		64.1 ± 19.3	203.1 ± 113.8	16.0 ± 3.6	10.0 ± 2.7	55.1 ± 11.5	45.1 ± 6.9	1.7 ± 0.2

### Response to playback

*Molossus molossus* activity was twice as high as *Saccopteryx bilineata* at our recording sites. We recorded 1–16 *M*. *molossus* passes during 116 of the 308 two-min playback sequences (Table 2), and 1–9 *S*. *bilineata* passes during 59 playback sequences. Overall, we recorded 154 *M*. *molossus* passes during distress call playback periods and 90 passes during the paired silent periods. During noise playback and paired silent periods, we recorded 76 and 90 passes, respectively. For *S*. *bilineata*, there were 58 and 36 passes during distress call and paired silent periods, respectively. During the noise playback and paired silent periods, we recorded 28 and 54 *S*. *bilineata* passes, respectively.

**Table 2:** Number of playback sequence leading to more or fewer passes.

Bat response	*M*. *molossus* distress call playbacks	Pink noise playbacks
*M*. *molossus*		
Passes increase	39	15
No change	8	12
Passes decrease	18	24
*S*. *bilineata*		
Passes increase	18	5
No change	4	4
Passes decrease	11	17

**Fig 3:**
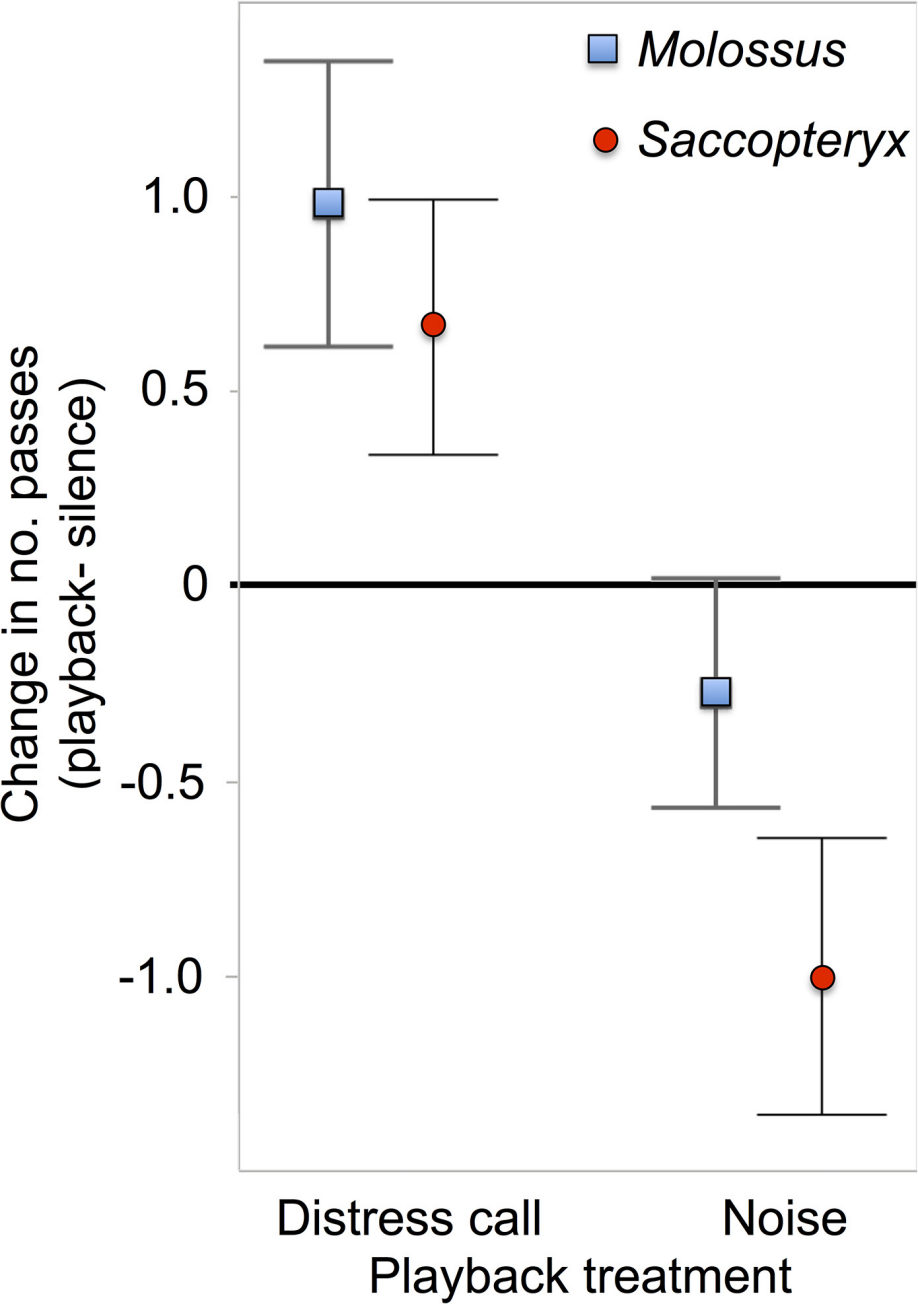
Effect of playback treatment on bat activity. The mean responsiveness (number of passes during playback period–silent period) is shown for *M*. *molossus* (blue squares) and *S*. *bilineata* (red circles) in response to either *M*. *molossus* distress calls or pink noise. Errors bars show standard error of the mean.

**Fig 4:**
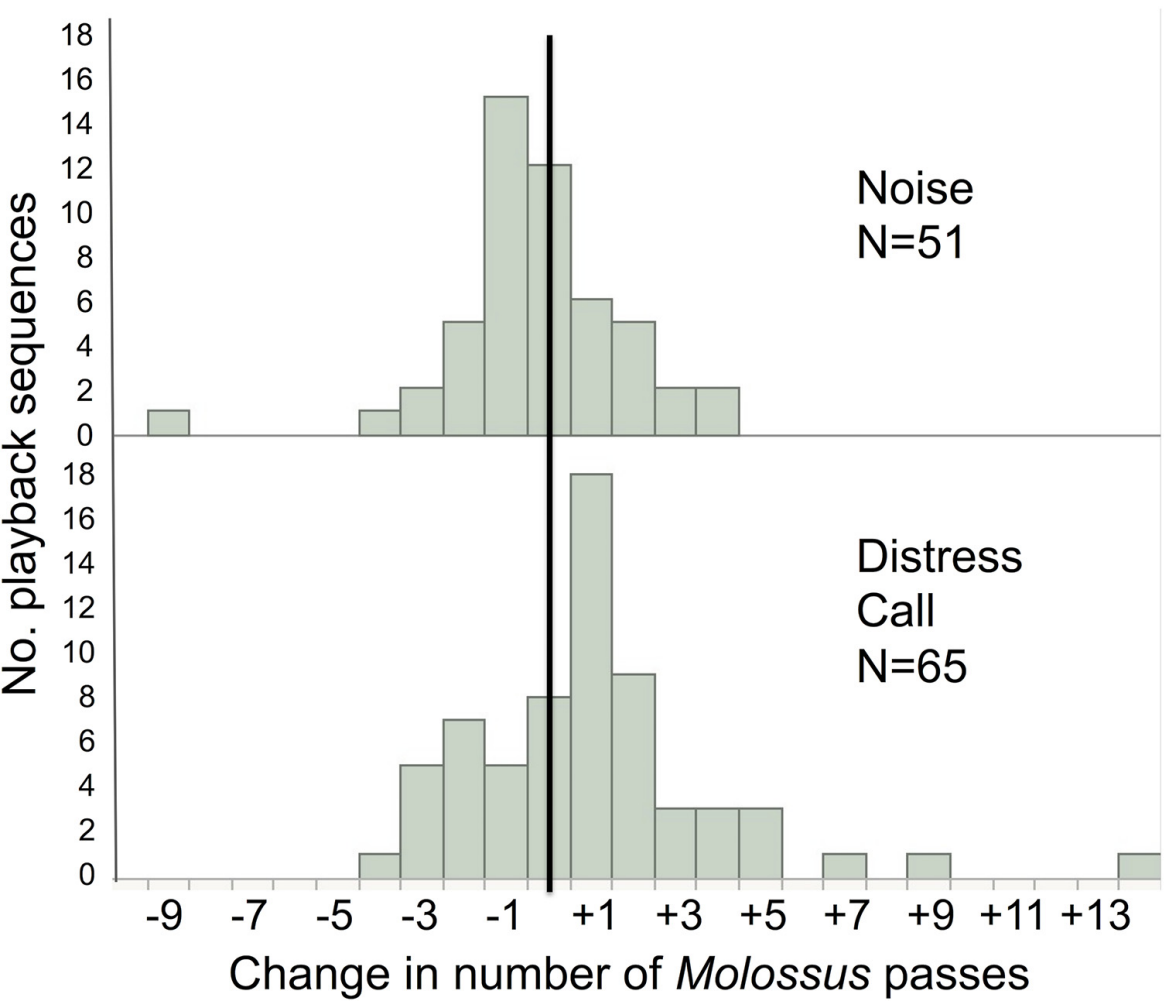
Distributions of *Molossus molossus* responsiveness. Frequency histograms of responsiveness during playback sequences that had passes of *M*. *molossus* (n = 116). Increases in activity are to the right of the solid line.

**Fig 5:**
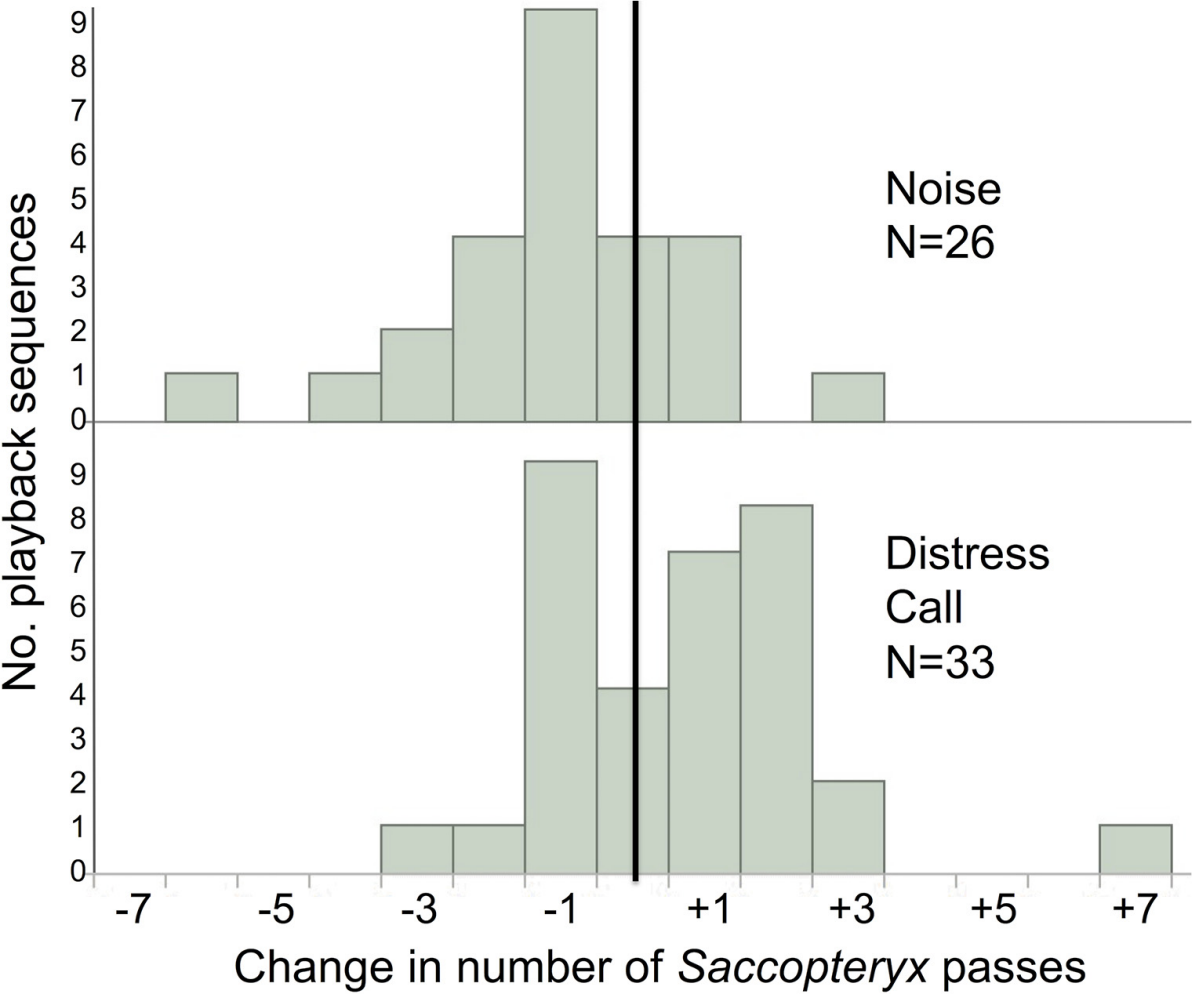
Distributions of *Saccopteryx bilineata* responsiveness. Frequency histograms of responsiveness during playback sequences that had passes of *S*. *bilineata* (n = 59). Increases in activity are to the right of the solid line.

*M*. *molossus* activity increased more during distress call playbacks than during noise playbacks (F(1,20.12) = 4.47, p = 0.0298: Fig 3), and this difference was driven by attraction to distress calls (t = 2.68, df = 64, one-sided p = 0.0047) rather than avoidance of noise (t = 0.93, df = 50, one-sided p = 0.16; Fig 4). Mean *S*. *bilineata* activity also increased more during playbacks of *M*. *molossus* distress calls compared to noise (F(1,16.79) = 14.95, p = 0.0013; Fig 3), and this difference was driven by both an attraction to distress calls (t = 2.04, df = 32, one-sided p = 0.0251) and an avoidance of noise (t = 2.82, df = 25, one-sided p = 0.0047; Fig 5). We observed passes by other unidentified bat species in only 20 of 308 playback sequences, and found no effect of playback on this limited activity (F(1,22) = 0.39, p = 0.5).

Social calls during passes by *M*. *molossus* occurred in only 17 of 308 playback sequences. During distress call sequences, passing *M*. *molossus* produced 3 social calls during silent periods and 9 social calls during distress call playback. During noise sequences, the 8 social calls were evenly divided between silent and noise periods. With this small sample size, we failed to detect an effect of playback treatment (t = 1.1, df = 15, p = 0.29) or a difference in social calling between distress call playback periods and silent periods (t = 1.96, df = 9, p = 0.08; 95% confidence interval of distress call playback effect on social call production = -0.09 to +1.29 social calls).

### Behavior of passing bats during periods of high responsiveness

Bats approached the speaker during distress calls, but they did not approach closely, and if they ever approached in groups they did so either rarely or not at all. Across 308 playback sequences, there were only 11 sequences that led to responsiveness of >3 passes. For *M*. *molossus*, this occurred during two noise sequences and 9 distress call sequences. However, in the video recordings we saw no visible evidence of approaches to within 1 meter of the speaker, either as individuals or in groups. If bats were inspecting the location for predation risk, they did so primarily as individuals. Single *M*. *molossus* passes consisted of fast straight flights that did not approach the speaker. In *S*. *bilineata*, responsiveness exceeded 3 passes in only one case where an individual (probably a territorial male) circled very closely around the speakers, but we did not see multiple *S*. *bilineata* making close approaches.

### *Molossus molossus* activity and responsiveness over time

**Fig 6:**
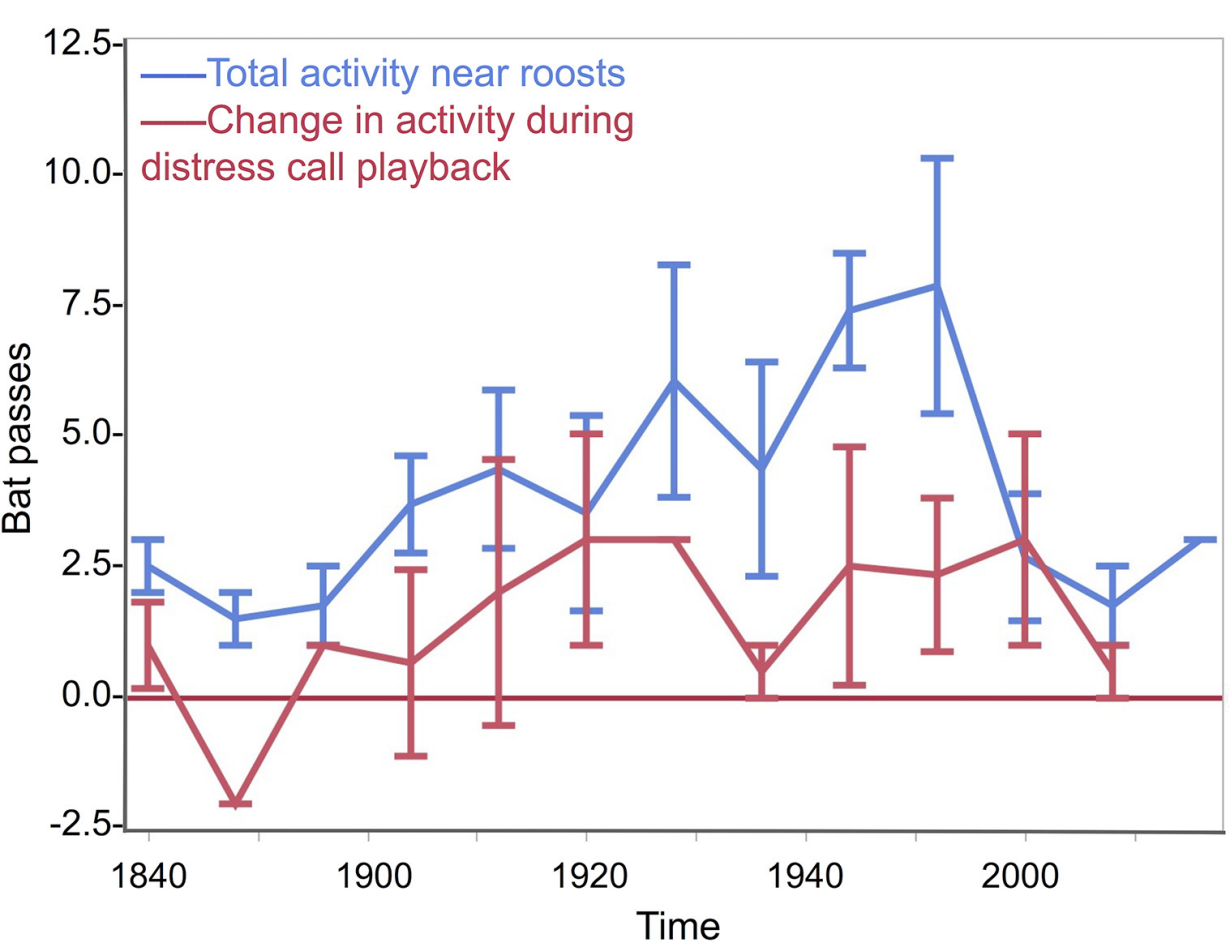
*Molossus molossus* activity and responsiveness to distress call playback near roosts over time. Mean number of passes (and standard errors) during the 2-min playback sequences recorded on four evenings from 1838 to 2020 h. Data are pooled in 8-min time bins (2–6 counts per bin). Counts do not include observations of zero passes. Blue line shows the total number of *M*. *molossus* passes during both distress call and noise playbacks. Red line shows the responsiveness (number of passes during the 1-min playback period minus 1-min silent period) during distress call playbacks.

*M*. *molossus* passes near roosts increased steadily after emergence at sunset then declined rapidly after about 1.5 h (Fig 6). Starting at emergence at 1840 h, activity increased until 2000 h (R^2^ = 0.28, F(1,44) = 17.02, p = 0.0002); but we found no evidence that distress call responsiveness increased over this same time period (R^2^ = 0.07, F(1,23) = 1.64, p = 0.21; Fig 6).

## Discussion

### Response to playback

Both the free-tailed bats *Molossus molossus* and sac-winged bats *Saccopteryx bilineata* were more attracted to the *M*. *molossus* distress call playbacks than to playbacks of silence or pink noise. *S*. *bilineata* also actively avoided the noise playbacks. The attraction of both bat species to distress calls cannot therefore be explained as a general investigation of broadband sounds of similar duration. The increase in activity in response to the *M*. *molossus* distress calls was larger for conspecifics than for *S*. *bilineata*.

The effect of distress call playbacks on bat activity was more consistent with individual inspection flights than mobbing by individuals or groups. *M*. *molossus* most often responded to distress calls with one additional pass (+1 pass after silence; +1.26 passes compared to noise); multiple passes during the 1-min playback were rare (Fig 4). Video analyses suggested that multiple passes were more likely to represent a single bat circling than a group of bats.

Why did *S*. *bilineata* respond to *M*. *molossus* distress calls? Several species of bats are known to respond to heterospecific distress calls [12, 15], and tests on the response of European pipistrelles to experimentally synthesized or modified distress calls show that some bats will respond to a range of conspecific-like distress calls depending on the spectral and temporal call characteristics [16]. Therefore, heterospecific bats may be generally responsive to such distress calls and *S*. *bilineata* were simply the most commonly detected heterospecific bats at our study site. Alternatively, responses from this species might have only occurred because individuals of these two species roost nearby each other at this site and therefore face shared predation threats. In either case, the pattern of responses by *S*. *bilineata* was also more consistent with individual inspections than cooperative mobbing.

Our findings should be considered with two limitations in mind. First, our results do not prove that mobbing does not occur or would not occur in this species under different circumstances. For example, bats may have reacted differently if we presented a life-like predator model. Second, responses may have differed if we played the distress calls of females rather than males. It is unclear if adult bat distress calls convey the sex or individual identity of callers, but males are more likely to produce distress calls in at least some species (e.g. *Sturnira lilium* [17]) and social relationships in most bat species are female-biased [45]. Finally, the hypothesis that distress calls promote mobbing in other bat species is not altogether unlikely given previous evidence of mobbing [30–31] and other similar cooperative behaviors in at least some bats [45]. However, future studies should examine the mobbing hypothesis more critically.

### Activity and responsiveness over time

In our study, *M*. *molossus* activity near roosts increased as the evening progressed from sunset until the bats returned to roosts after their first foraging bout. However, we failed to detect an increase over time in responsiveness (Fig 6). This may have been due to the large variance (range: -2 to +9 passes) and small sample (n = 25) of distress call responsiveness values, but it is also possible that bats are just as responsive to predation risk when departing from roosts as when returning to roosts. This finding is also more consistent with the predator inspection hypothesis than the mobbing hypothesis.

### Distress call structure and function

Many authors have proposed non-mutually-exclusive functions of distress calls in vertebrates, but the fitness benefits of distress calls remain ambiguous [1–23]. Russ et al. [14–15] concluded that distress calls by pipistrelles likely function to attract conspecifics that perform mobbing behavior. Unlike pipistrelles, however, the poor maneuverability of *M*. *molossus* could severely limit their ability to perform flight patterns needed for effective mobbing, such as tight circling within a cluttered environment. We observed no mobbing behaviors or close approaches to the speaker during the times when we recorded a large increase in *M*. *molossus* passes. Instead, bats typically flew by quickly in straight paths. These observations combined with the relatively weak response of conspecifics compared to pipistrelles [14–15], and the observation that highly maneuverable *S*. *bilineata* approached and circles the speakers more closely than *M*. *molossus*, suggest that *M*. *molossus* distress calling does not promote conspecific mobbing behavior.

A simpler adaptive explanation is that captured bats produce distress calls to startle naive or inexperienced predators into releasing the caller [6–8, 17, 23]. This startle hypothesis is consistent with the presence of nonlinearities, such as sideband modulations [43], and a large variation in call structure both among and within callers, which can reduce habituation [46–50]. Russ et al. [14] argued that pipistrelle distress calls would not be effective at startling avian predators because the lower frequency limit of these calls is about 17 kHz, which is beyond the audible range of raptors and owls (up to 10–12.5 kHz). In contrast, *M*. *molossus* distress calls have a mean lower frequency limit of 10 kHz and could more easily serve as signals to both avian and mammalian predators. It would be interesting to see if *M*. *molossus* distress calls are structurally similar to calls given in agonistic contexts, because the startle hypothesis predicts that calls produced in either context should provide the same information regarding the ability of the caller to attack or defend itself. For example, avian distress calls contain information about health and thus ability to defend or escape [8].

Distress call structure varies across bat species, but common design features include longer durations, lower frequencies, and greater bandwidth relative to other social or echolocation calls [17]. These general acoustic characteristics do not implicate a single clear function because the same design might reflect multiple possible selective pressures, such as maximizing travel distance, conveying honest information about the caller’s ability to defend itself, or ensuring the signal is heard by a broadest range of predators. The potential fitness benefits of distress calls are also difficult to confirm because predation events are rare but have extreme fitness consequences. A single escape, however unlikely, could easily outweigh the fitness costs of being a frequent screamer when captured. That is, even if the success of mobbing, startling, or attracting other predators, is very unlikely—these rare positive outcomes may still provide an adequate benefit to maintain the trait of distress calling over evolutionary time.

Regardless of the adaptive function of distress calls, the most parsimonious explanation for the response of both conspecifics and heterospecifics is that approaching bystanders are merely investigating sources of distress calls to gather information about predation risk. Our results suggest that mobbing in response to male *M*. *molossus* distress calls is rare or nonexistent. *M*. *molossus* distress calls likely have other functions because this species frequently produces distress calls (even more so than many other bats), but does not appear to cooperatively mob in response to them (even less so than other bats). Based on our results, we suggest that the observation of bats approaching distress call playbacks does not itself provide clear evidence for mobbing. Future studies seeking to test the mobbing hypothesis would benefit from playing calls of both familiar and unfamiliar conspecifics of both sexes, and presenting visible life-like predator models, e.g. [51].
